# Community structure and methylation of microbes in an artificially forced sediment core

**DOI:** 10.1128/spectrum.03533-25

**Published:** 2026-05-28

**Authors:** Rui Zhao, Jennifer F. Biddle

**Affiliations:** 1 School of Marine Science and Policy, University of Delaware 5972, Lewes, Delaware, USA; Oliveira, CEA-Genoscope, Evry, France

**Keywords:** sediment, epigenetics, methylation, metagenome

## Abstract

Epigenetic modifications, such as DNA methylation, may be used in prokaryotes for the adaptation of microbes to external environmental changes. In this study, we examined the microbial community structure, recovered the genomes of the dominant microbes, and tracked methylation in several dominant microbes in a 23-cm artificial sediment core formed in a settling tank that mimics the sediment formation process. Our results indicated that the prokaryotic communities only showed minor variations with depth and were dominated by bacteria (especially taxa of Deltaproteobacteria, Gammaproteobacteria, and Bacteroidota), while archaea (dominated by Bathyarchaeia) accounted for <5% of the total communities throughout the core. We detected methylation by analyzing metagenome sequencing data of methyl-specific enzyme-digested and undigested DNA. We recovered 72 high- or medium-quality metagenome-assembled genomes for the dominant taxa, for 7 of which we detected distinct downcore methylation patterns. This work highlights the diverse processes of epigenetic modification in response to the sediment burial process, which may have a long-term impact on the overall community fitness in the evolving energy-limited conditions in marine sediments.

**IMPORTANCE:**

This work reports changes in the epigenetic profiles of microbes buried in a sediment column formed under a controlled, artificially created environment. This approach removes confounding variables of bioturbation and changes in sediment flux. We also use an approach that is accessible for low amounts of DNA to determine methylation status.

## INTRODUCTION

Most prokaryotes in natural environments live with much lower energy per cell than those measured in the laboratory (1–3). The majority of these prokaryotic cells reside in the deep subsurface (4), where the energy density generally decreases with sediment depth and age (5). They are thought to persist under these energy-limited conditions with specific adaptations (6) and negligible genomic evolution (7), although some of them are suggested to show net growth in their ideal niche with elevated power supplies (8–10). However, except for a few cases (e.g., references 11–16), most of the dominant microbes in marine subseafloor sediments remained uncultured (17), and to date, the adaptation mechanisms of microbes still remain mysterious.

DNA methylation, a form of epigenetic modification catalyzed by DNA methyltransferases (MTases), is a widespread phenomenon in all domains of life (i.e., in bacteria and archaea [18] and also viruses [19, 20]) and may play a role in microbes adapting to energy-limited environments (21). Prokaryotic DNA contains three types of methylation: N6-methyladenine (6mA), N4-methylcytosine, and 5-methylcytosine (5mC), among which 6mA is the most prevalent (22). The biological roles of DNA methylation are most extensively studied in mammals, where it contributes to normal development and disease via its impact on gene expression (23, 24). In bacteria, DNA methylation is best known for its role in restriction-modification (R-M) systems that are thought to provide defense against phage infection and limit horizontal gene transfer through the degradation of invading non-methylated DNA (25). It is worth noting that a substantial fraction of MTases detected in prokaryotes are orphan and unexplored MTases (18, 20), which do not belong to R-M systems, and their biological functions have been suggested to be involved in gene expression regulation (26, 27), DNA replication (28), DNA repair (29), and cell cycle regulation (30). Because these processes are critical for the fate (thriving, maintaining, or decaying) of microbes in the environment, DNA methylation may exert great influence on the adaptation of microbes to external environment changes, such as decreasing energy availability. Our current knowledge of prokaryotic epigenomics mostly relies on studies with culturable strains (e.g., *Escherichia coli* [31], *Geobacter sulfurreducens* [32], and Cyanobacteria [33], among others). The study of methylation of microbes in the environment is growing but still understudied in comparison to cultures (20, 21).

To study the methylated motifs of microbes in the vast uncultured microbes inhabiting natural environments, genome-resolved metagenomics and methylation detection by high-throughput sequencing have been applied for freshwater and marine environments (20, 34). Additionally, bulk metagenome characterization following restriction enzyme treatment has also been employed to study the dynamic methylation profiles of the bulk microbial community in estuary sediment spanning over 100 years (21). These previous studies demonstrated that it is possible to characterize the epigenetic modification of microbes in response to external changes in the natural environments via high-throughput sequencing (35).

Although the single-molecule, real-time sequencing using PacBio or Nanopore platforms is regarded as the gold standard for methylation characterization (22), these approaches require high DNA input (>1 µg) that is challenging to obtain from natural samples such as marine sediments, particularly in biogeochemically relevant horizons. Therefore, we chose a methylation-analysis approach of restriction enzyme digest, followed by short-read next-generation sequencing. This approach can be used for experimental validation of methylated sites, is highly sensitive, and requires limited input DNA (22). The enzyme-cleaved DNA will preferentially survive library creation and create an imbalance of assembled reads that are statistically significant (21). To further study the role of DNA methylation in the adaptation of microbes to energy-limited marine sediments, we extended the previous work of Rambo et al. (21) that examined the dynamics of methylation in the bulk sediment community. Instead of studying naturally formed sediments spanning over 100 years (21), we focused on an artificial sediment core formed in the laboratory, which represents an extreme case with a short settling distance (<60 cm) and therefore rapid sedimentation and fast microbial responses. The core was formed in about 9 months (16 November 2016–21 August 2017). We hypothesize that this experimental setup could reproduce the sedimentation process in a controlled manner, allowing us to observe microbial responses through a multidisciplinary lens in a much shorter time frame. We examined the overall community structure and identified the dominant microbes by 16S rRNA gene amplicon sequencing. We then performed metagenome sequencing, assembly, and binning to obtain draft genomes for the dominant microbes in the samples. Our concurrent methylome sequencing data also allowed the detection of methylated motifs in these dominant genomes. Our study indicates that microbial communities residing in the artificial core formed during the short-term settling experiment with high sedimentation rates exhibited far smaller downcore community composition variations than those frequently seen in natural marine systems. Despite these, some of the dominant microbes still exhibit variable methylation responses to the sediment accumulation process.

## MATERIALS AND METHODS

### Setup of the sediment-settling experiment

**Fig 1:**
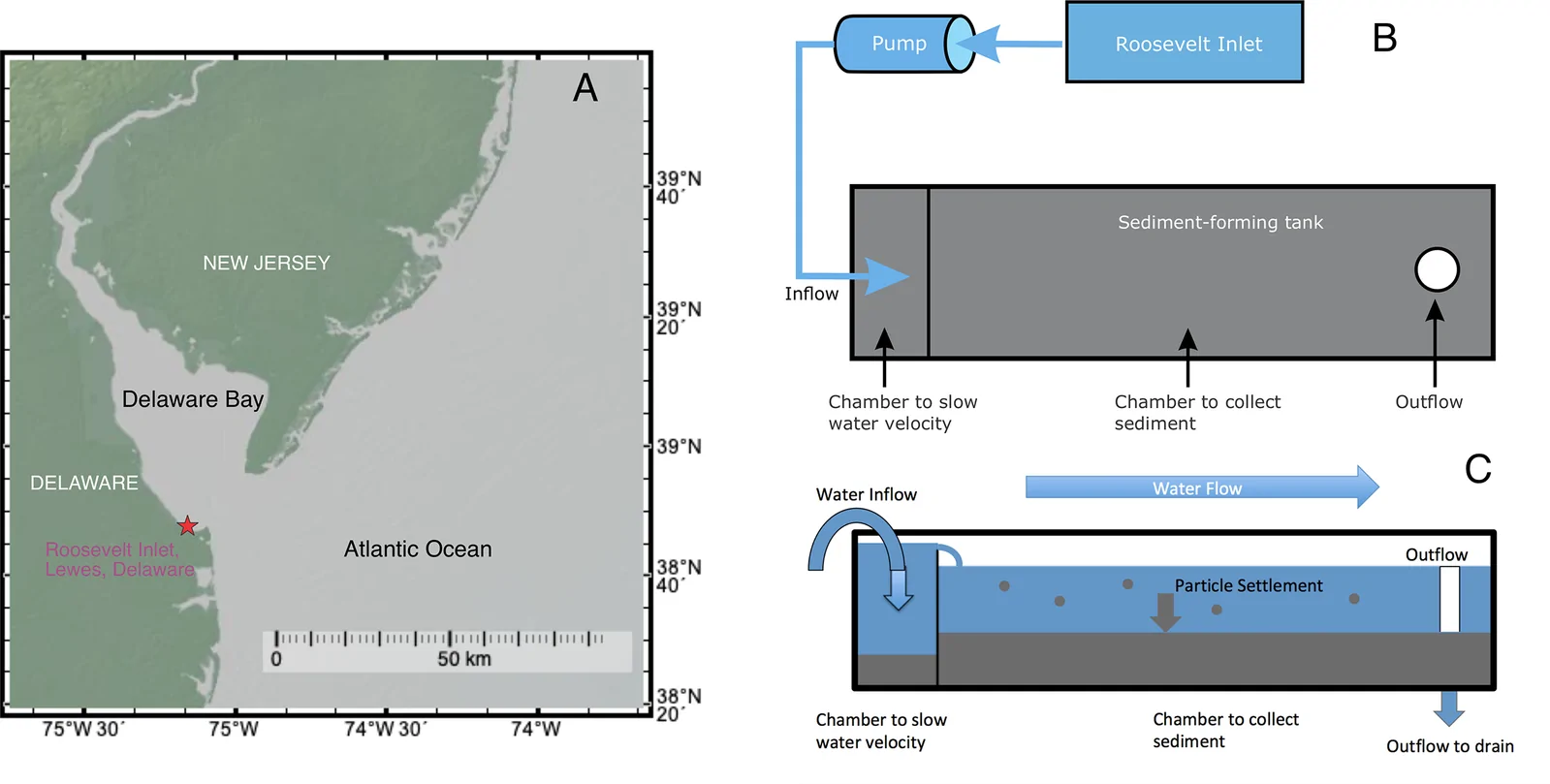
Sampling location and the setup of the sediment-forming experiment. (**A**) A map showing the location of the Roosevelt Inlet on the coast of Delaware, USA, where the seawater was used as the inflow of our sediment-forming experiment. The map is prepared using the default basemap (37) of GeoMapApp (http://www.geomapapp.org). (**B**) A diagram showing the setup of the sediment-forming experiment, in which the inflow water was introduced into the tank by a pump. The water was first pumped into a narrow chamber to decrease the velocity to minimize the disturbance potential caused by water flow. (**C**) The cross-sectional view of the same setup.

The sediment formation process was mimicked by a designed sediment tank such that flowing, unfiltered seawater could be pumped at low velocity and trapped for a time period in the tank, allowing entrained particulate matter to settle out of the water column. A detailed description of the design is provided in reference 36 and is shown in Fig. 1. Briefly, incoming water was taken as unfiltered seawater pumped directly from the Roosevelt Inlet of the Delaware Bay in Lewes, DE (38°47′29.04″ N, 75°9′54.82″ W, Fig. 1A) and into a chamber at the front of the tank. The water inflow was first introduced into a narrow, high comber to decrease the water velocity. This can minimize the sediment disturbance caused by the inflow. Then, the water was trickled into the main, sediment-forming chamber. An elevated outflow at the back end of the main chamber allowed excess water to drain and directed the flow of water across the tank (Fig. 1B and C). The system is distinct from natural systems in that filter feeders (primarily tunicates) were intentionally removed and that very few bioturbators entered the system, both of which may significantly impact community dynamics in natural sediments. This experimental design allowed isolating particle settlement in order to directly investigate how this process impacts continuous sedimentary microbial community development from water column particulate matter.

For this study, a 23-cm long sediment core formed between 16 November 2016 and 21 August 2017 was retrieved from the sediment-forming chamber using a 10-cm-diameter plastic corer. The sediment core was gradually extruded from the corer for 23 steps. At each step, 1 cm of sediment was pushed out and transferred into a sterile plastic bag using a stainless steel plate. In this way, the core was evenly divided into 23 layers of 1 cm thickness. The sample bags were preserved at −80°C for further analysis.

### DNA extraction

Total DNA was extracted from 0.5 g of sediment. Prior to the extractions, extracellular DNA was removed following the recommendation of Lever et al. (38) to minimize the impact of relic DNA in the samples. Briefly, the samples were incubated for 1 hour in the first round with a carbon dissolution matrix (0.43 M acetic acid and 0.43 M sodium acetate [pH 4.7]) and 1 hour in the second round after adding 10× TE buffer (300 mM Tris-HCl and 10 mM EDTA [pH 10.0]). All incubations were done at room temperature on a shaker incubator (600 rpm). After incubations, samples were centrifuged for 20 min at 10,000 × *g* to remove extracellular DNA in the supernatant. The pellets were retained for DNA extraction using the DNeasy PowerSoil DNA Isolation Kit (QIAGEN, Hilden, Germany) following the manufacturer’s instructions. The nucleic acids were finally eluted into 100 µL ddH_2_O. After the extraction, DNA concentration was quantified by Qubit 3.0 and NanoDrop 2000. The DNA was preserved at −20°C for subsequent 16S rRNA gene amplicon sequencing, qPCR analyses, methylation sequencing, and metagenome sequencing.

#### Quantitative PCR

The bacterial and archaeal 16S rRNA ribosomal genes were quantified using the primer sets Uni341/Uni519 and Uni515F/Arc908r combined with the thermal conditions described in reference 39. All qPCR reactions were run in triplicate, and each reaction mixture contained 1× QuantiTect SYBR Green PCR Master Mixture (QIAGEN, Hilden, Germany), 0.5 μM forward and reverse primers, and 1 μL of DNA template in a final volume of 20 μL. The standard for each gene was linear genomic DNA from *E. coli* (bacterial 16S rRNA gene) or *Methanosarcina mazei* (archaeal 16S rRNA gene). For each gene, the DNA concentration of the standard was measured using Qubit, and a DNA abundance gradient of 10–10^8^ copies µL^−1^ was prepared by 10× serial dilution. Gene abundances were normalized to copies per gram sediment.

### Preparation for 16S rRNA gene amplicon sequencing and sequence analysis

A two-step strategy was employed to prepare the amplicons of the V4 region of the 16S rRNA gene with the primer pair Uni519F/806R and thermal cycling conditions described elsewhere (39). Because the initial, undigested DNA concentration was similar across all samples, in the first-round PCR, 1 µL of DNA template and 27 PCR cycles were applied for all samples to obtain similar amounts of PCR products. Duplicate PCR reactions were pooled and purified using the GenElute DNA Purification Kit (Sigma-Aldrich, St. Louis, MO, USA). In the second-round PCR of seven cycles, barcodes were attached to the primer with the same nucleotide sequences as the first-round ones. Concentrations of PCR products after purification were measured with Qubit 3.0 (Thermo Fisher Scientific, Waltham, MA, USA), and equal amounts of DNA from all samples were pooled. The amplicons were sequenced (2 × 150 bp paired-ends) on an Illumina HiSeq 2000 sequencing platform (Illumina, San Diego, CA, USA) at the University of Connecticut.

Sequencing reads were quality filtered and trimmed using USEARCH v11 (40), and chimeras were detected and removed using UCHIME (41). Paired-end reads were merged (287 bp) using USEARCH (40). Trimmed reads were *de novo* clustered into operational taxonomy units (OTUs) at >97% nucleotide sequence identity using UPARSE (40). Samples were subsampled to 27,000 reads for each sediment horizon with the –otutab-norm command in USEARCH v11 (40). The taxonomic classification of OTUs was performed using the lowest common ancestor algorithm implemented in the CREST v4 package (42), with the SILVA 138.1 release as the reference. In the final visualization in bar charts, phyla accounting for <0.2% (<54 reads) in any sample were aggregated into “Others.”

### DNA methylation analysis and sequencing

An aliquot of the extracted DNA (i.e., the DNA extracted from 0.5 g sediment post-extracellular DNA removal) was treated as described in reference 21 for the methylation analysis. A 10 µg aliquot of purified gDNA was digested with the methylation-sensitive restriction endonuclease HpaII, which cleaves unmodified CCGG sites and leaves C-5mC-GG sites intact, assisting in the recognition of methylated 5′ cytosine (m^5^C). The restriction enzyme approach introduces a methylation-dependent fragment distribution into the gDNA library, which significantly enriches the presence of methylated motifs in metagenomes (21). Digested DNA was cleaned with a QIAquick PCR Purification Kit (QIAGEN, Hilden, Germany) and subsequently digested with the methylation-sensitive restriction enzyme DpnII, which cleaves unmodified GATC and leaves G-6mA-TC sites intact, allowing for the detection of N6-methyladenosine (m^6^A). DNA was then cleaned via the PCR purification kit again and sent for DNA sequencing. Samples were sheared to a median size of 300 bp using a Covaris focused-ultrasonicator, and Illumina libraries were prepared using the NEBNext Ultra Library Prep Kit for Illumina (New England BioLabs, Ipswich, MA, USA) and sequenced with an Illumina HiSeq 2500 at the University of Delaware Sequencing and Genotyping Center (Newark, DE, USA). Single-read sequencing was performed for all samples, with a read length of 76 bases.

### Metagenome assembly, genome binning, and refinement

Undigested DNA was also sent to the UD Sequencing and Genotyping Center for metagenome sequencing. Metagenomic libraries were prepared and sequenced on an Illumina NextSeq 500 sequencing platform (Illumina, San Diego, CA, USA), with paired reads at a length of 251 bases. Quality of the reads and presence of adaptor sequences were checked using FastQC v0.11.5 (43). Then, the sequencing data were processed with Trimmomatic v0.36 (44) to trim read-through adapters (ILLUMINACLIP:TruSeq2-PE.fasta:2:30:10), trim low-quality base calls at the starts and ends of reads (LEADING:3, TRAILLING:3), remove reads that had an average Phred score lower than 25 in a sliding window of 10 bp (SLIDINGWINDOW:10:25), and finally remove reads shorter than 100 bp (MINLEN:100). The overall quality of processed reads was evaluated in a final check with FastQC v0.11.5 (43) to ensure only high-quality reads were used in the downstream analysis.

Metagenome assembly, genome binning, and refinement were performed following previous descriptions in reference 45. The quality-controlled paired-end reads were *de novo* assembled into contigs using Megahit v1.1.2 (46), with the k-mer length varying from 27 to 117. Contigs larger than 1,000 bp were grouped into genome bins with MaxBin2 v2.2.6 (47) using the default parameters. The quality of the obtained genome bins was assessed using CheckM2 v2.1.1 (48). Genome bins of >50% completeness were manually refined using the R package *gbtools* (49), based on the GC content, taxonomic assignments, and genome coverages in multiple samples. Coverages of contigs in each sample were determined by mapping trimmed reads onto the contigs using BBMap v37.61 (50). Taxonomy of contigs was assigned according to the taxonomy of the single-copy marker genes in contigs identified using a script modified from blobology (51) and classified by BLASTn (52). SSU rRNA sequences in contigs were identified using Barrnap (53) and classified using VSEARCH (54). To improve the quality of metagenome-assembled genomes (MAGs), metagenome reads from the sample with the highest coverage were aligned to the MAG contigs using BBmap (50). Then, the aligned reads were re-assembled using SPAdes v3.12.0 (55) with the default parameters and a minimum contig length of 1,000 bp. The resulting scaffolds were visualized and re-binned using *gbtools* (49) as described above. The qualities of the resulting MAGs were assessed using CheckM2 v2.1.1 again.

### Genome classification and annotation

All genomes were classified using GTDB-Tk v2.2.6 (56), with GTDB Release 10-RS226 as the reference database. Genome annotation was conducted using Prokka v1.13 (57), eggNOG (58), and BlastKoala (59) using the KEGG database. The functional assignments of genes of interest were also confirmed using BLASTp against the NCBI RefSeq database.

Average nucleotide identity between different genomes was calculated using FastANI with default parameters (60). Average amino acid identity (AAI) was calculated using CompareM (https://github.com/dparks1134/CompareM) with the “aai_wf” option, in which the protein coding sequences predicted by Prodigal (61) were used as input to identify orthologous proteins by an all-vs-all reciprocal sequence similarity search with Diamond (62). The average similarity of the orthologous proteins between the two genomes was taken as the pairwise AAI.

### Methylation sequencing analysis

Cytosine and adenosine methylation were calculated using a commercial bioinformatic pipeline to reconstruct probabilities of methylation at CpG and ApT sites ([63, 64] Genome Profiling LLC, Newark, DE, USA), as described previously (21). Briefly, we compared the assembly metrics of undigested and digested DNA and used the statistical probability of uneven read coverage based on digestion sites to estimate the probability of base methylation.

## RESULTS AND DISCUSSION

### Sediments formed under controlled conditions

For this study, we focused on a sediment core formed in a sediment-settling experiment, in which the sediments were deposited under controlled conditions: (i) high organic content in the incoming water, as evidenced by the 20–130 mg/L of chlorophyll *a* (36), and (ii) extremely short depositing depths (i.e., 60 cm as the height of the sediment tank) and therefore short oxygen exposure time during the settlement process (Fig. 1B and C). These variables are among the primary controlling factors of organic matter preservation in the seafloor (65), the combination of which is anticipated to result in high organic matter contents in the sediments. Consistent with this, oxygen in the formed sediments was measured to penetrate only 1 mm in the sediment column (36), presumably due to the high organic matter content. Therefore, the sediment layers investigated in this study were anoxic.

The sediment core BRC2 is 23 cm long and was formed in 278 days. On average, the sedimentation rate was estimated to be 30.2 cm/year, which is about 200-fold higher than that of the nearby Delaware Bay (0.15 cm/year [66]) and a million-fold higher than that in the South Pacific Gyre (10^−5^–10^−4^ m/year [67]), one of the slowest sediment accumulation sites on Earth.

#### Abundances of bacteria and archaea in core BRC2

**Fig 2:**
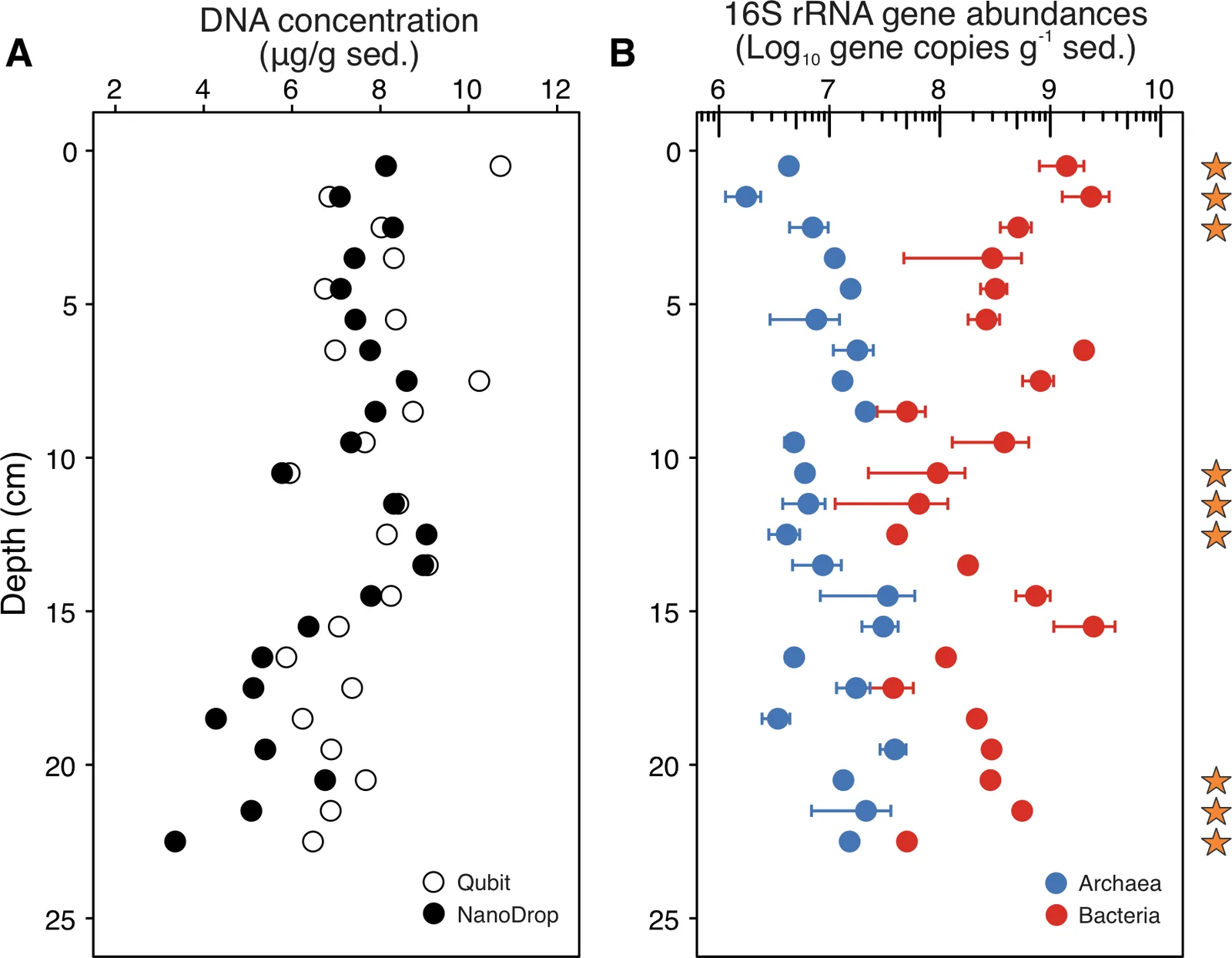
DNA concentration and abundance of archaeal and bacterial 16S rRNA genes in core BRC2. (**A**) DNA concentrations were measured using two different methods (i.e., Qubit and NanoDrop). (**B**) Abundances of the 16S rRNA genes were determined by qPCR using the domain-specific primers. The samples selected for metagenome sequencing are highlighted by stars.

To track the population dynamics of microbes during the sediment-settling experiment, we quantified the concentration of the extractable DNA from 23 sediment layers of core BRC2 (i.e., one sample per 1 cm of sediment). Our result indicates that the extractable DNA concentrations measured by two methods (i.e., Qubit and NanoDrop) generally matched with each other and were stable in the upper 14 cm but showed a sharp decrease below that depth (Fig. 2A), probably indicating microbial decay in the deeper energy-limited sediments. To check if archaea and bacteria have different responses to the sediment burial process, we quantified the abundances of archaea and bacteria throughout the core by qPCR using the domain-specific primers. Our results showed that while archaea generally increase with depth, the abundance of bacteria generally has a decreasing trend with depth, although local increases (e.g., ~7 cm and ~15 cm) were also detected (Fig. 2B). In addition, the prokaryotic community was dominated by bacteria, with archaea accounting for generally <10% of the total communities throughout the core.

#### Stable microbial community composition and structure along the sediment formation

**Fig 3:**
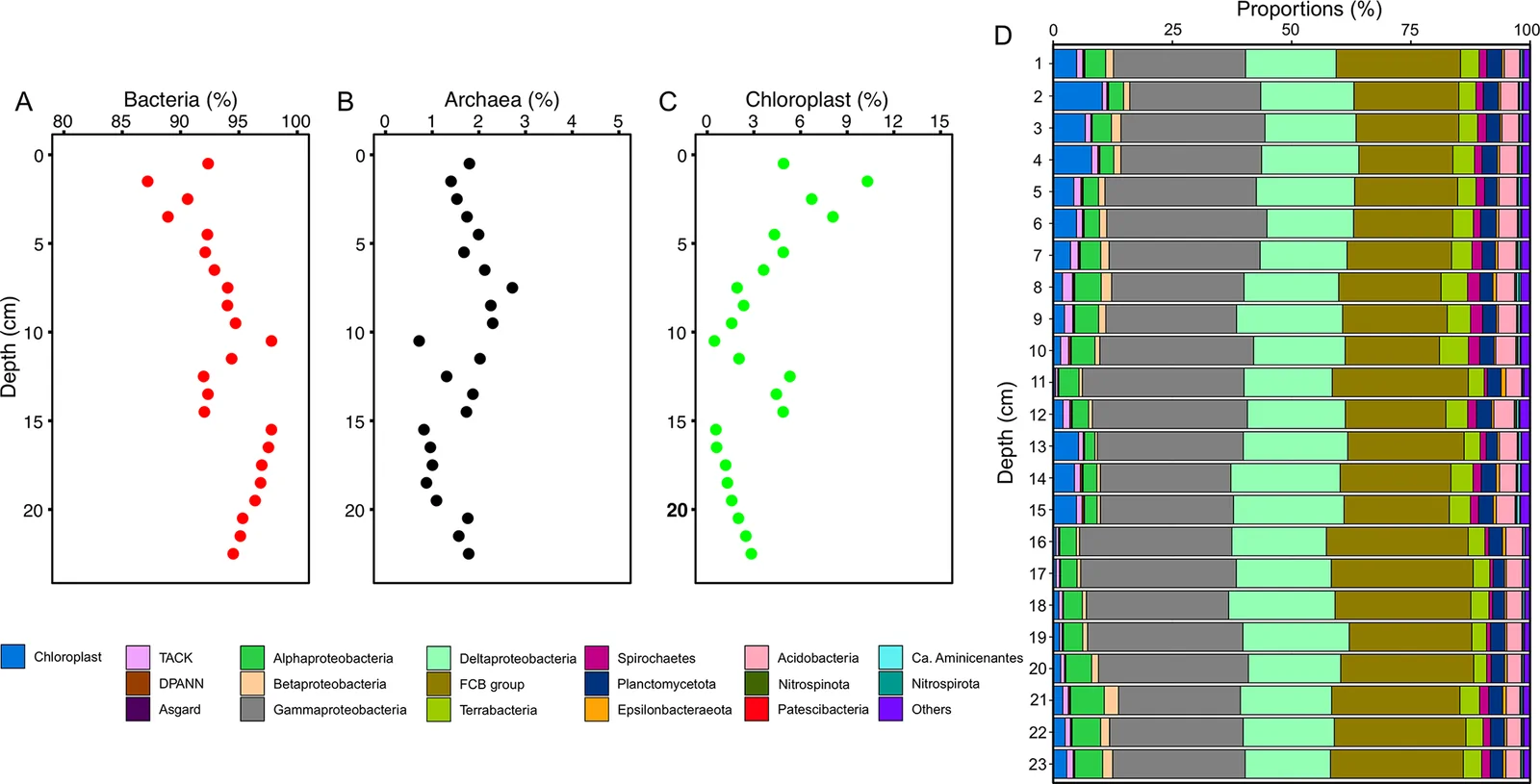
Downcore variation of the microbial communities in core BRC2. (**A–C**) Relative abundance of bacteria (**A**), archaea (**B**), and chloroplast (**C**) in core BRC2 as assessed by 16S rRNA gene amplicon sequencing. (**D**) Phylum-level community composition assessed by 16S rRNA gene amplicon sequencing.

To assess whether the overall microbial community varies through depth, we characterized the microbial community composition and structure by 16S rRNA gene amplicon sequencing. Consistent with the abundance quantification results from qPCR, the microbial communities were dominated by bacteria (Fig. 3), with archaea (dominated by Bathyarchaeota in the TACK superphylum) accounting for 0.5%–3.0% of the total communities in all depths (Fig. 3B). Overall, the bacterial communities were dominated by Gammaproteobacteria, Deltaproteobacteria, and Bacteroidota (formerly Bacteroidetes, corresponds to FCB group shown in Fig. 3), with little downcore variations on the bacterial phylum level (Fig. 3D). In addition, photosynthetic chloroplast was also detected throughout the core (Fig. 3C). The relative abundance of chloroplast in the total communities generally decreased with depth, although spikes were also detected in the middle (13–15 cm) section of the core (Fig. 3C). Assuming a constant sediment-forming rate, the 13–15 cm sediment interval is estimated to be formed in the spring (11 April–17 May) of 2017. Considering the often-observed spring bloom of phytoplankton in the Delaware River Estuary (68), the chloroplast spike probably resulted from the spring bloom of these photosynthetic organisms in the incoming water (i.e., the Roosevelt Inlet water).

The predominance of the same classes of Gammaproteobacteria, Deltaproteobacteria, and Bacteroidota was also detected in the incoming water (36), suggesting that the microbes detected in the sediments were mainly derived from particulate matter in the incoming seawater. The lack of stratification in the microbial community structure in the sediment core is contrary to observations in typical deep-sea sediments (e.g., references 69, 70). Sediment age and diagenetic state are key drivers of microbial community assembly in subsurface sediments (71). The rapid sedimentation rates in the lab sediment-settling tank may not leave sufficient time to allow the occurrence of organic matter diagenesis and microbial taxa selection, driving the prevalence of surface-dominating taxa in the subsurface sediments. The stable microbial community structure observed in the incoming water (36) may also contribute to the minor variations detected in the microbial community structures of the deep sediment layers.

#### Dominant microbes in the BRC core

**Fig 4:**
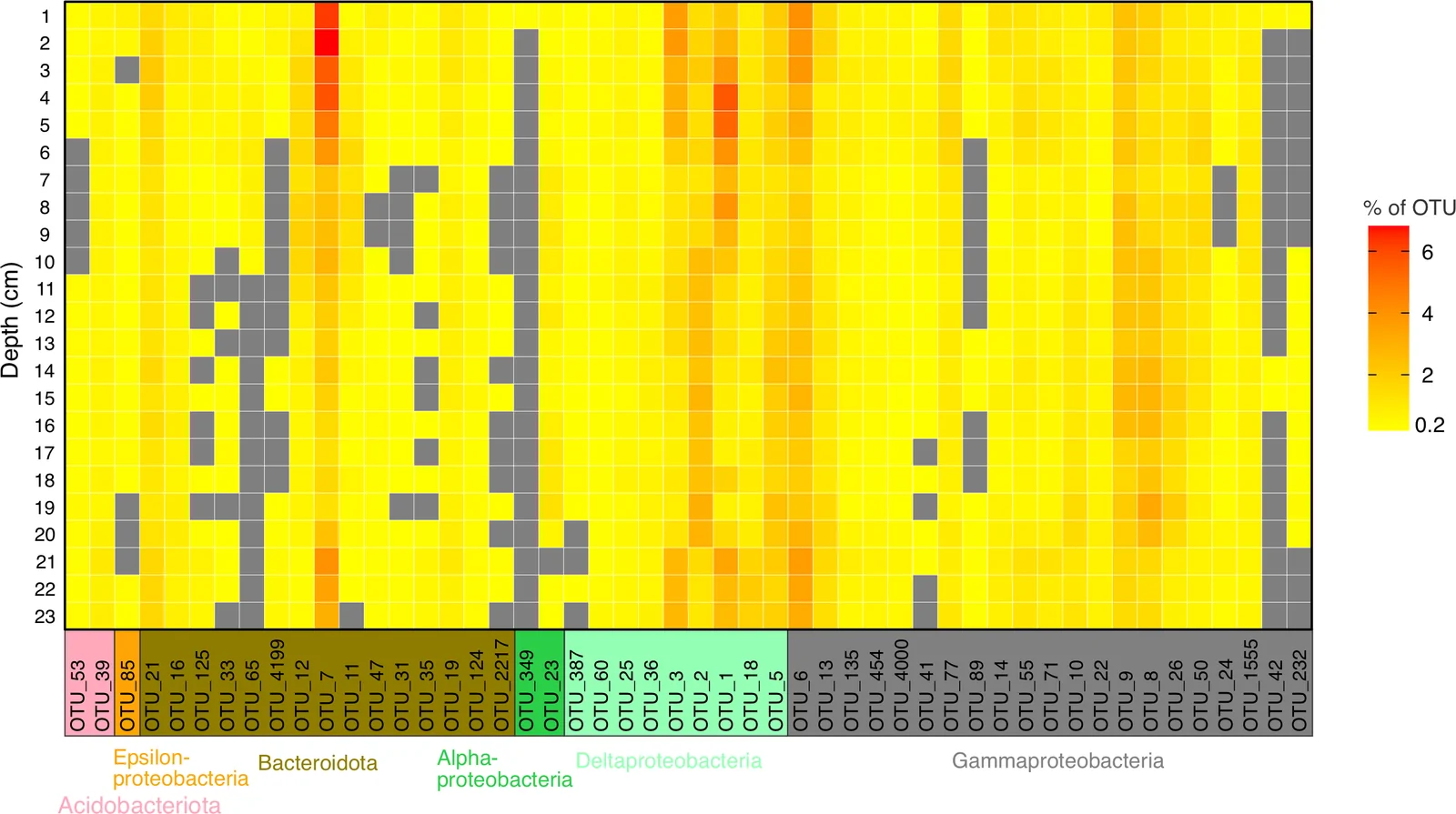
Heatmap showing the occurrence and relative abundances of the top 50 OTUs in core BRC2. Gray squares denote OTUs of absence or of <0.2% relative abundances. The OTUs are sorted based on their phylum-level taxonomy, which is also shown at the bottom.

To examine the identities of the dominant microbes in core BRC2, we looked more closely at the amplicon sequencing data on the individual OTU level. Overall, the microbial communities are of high diversity, with individual OTUs accounting for <7% in any layers (Fig. 4). The two most abundant OTUs are OTU_7 and OTU_6 (Fig. 4). A BLASTn search against the NCBI database suggested that OTU_6 is affiliated with the order Chromatiales (in the class Gammaproteobacteria), with *Wenzhouxiangella sediminis*, a chemoheterotrophic and facultative anaerobic bacterium isolated from tidal flat sediments (72), as the closest cultured representative. OTU_7 was affiliated with *Lutimonas* in the order Flavobacteriales (Bacteroidota phylum). The cultured relatives of OTU_7 include *Lutimonas vermicola* isolated from marine polychaete *Periserrula leucophryna* (73), *Lutimonas saemankumensis* SMK0142 (formerly *Aestuariicola saemankumensis*) isolated from tidal flat sediments (74), and *Lutimonas halocynthiae* isolated from a golden sea squirt (75). The dominance of *Lutimonas* in marine sediments was also observed in Black Sea sediments (76). In addition, OTU_1 is classified as a member of the Sva1033 family within the Desulfuromonadales order in Deltaproteobacteria. OTU_12 (the 22nd most abundant OTU) is affiliated with the *Lutibacter* genus in the Flavobacterales order within the Bacteroidota phylum, which should correspond to Bin_491. OTU_9 is classified as Woeseiaceae, a bacterial taxon widespread in tidal flat sediments (77), with *Woeseia oceani* as the cultured representative (16). These results suggest that the dominant microbes in core BRC2 formed in the sediment-settling experiment have cultured relatives, suggesting they are capable of fast, adaptive growth, even though their genomes have not been sequenced.

### Draft genomes of microbes dominating in core BRC2

To obtain the genomes of the above-described dominant microbes, we selected three sediment horizons from the top (#1–3), the middle (#11–13), and the bottom (#21–23) of core BRC2 for metagenome sequencing. From the co-assembly of the nine metagenome sequencing data, we obtained 72 MAGs of high or medium quality (Table S1). All recovered MAGs except Bin_175 are bacteria (Table S2), consistent with the dominance of bacteria in the total community as shown by the 16S rRNA gene amplicon sequencing and qPCR of 16S rRNA genes. Bin_175 is a MAG classified as Bathyarchaeota, whose dominance in nearby Broadkill River sediments has been documented previously (36). Among the bacterial MAGs, Bacteroidota (23/72), Gammaproteobacteria (26/72), and Myxococcota and Desulfobacterota (the latter two were from Deltaproteobacteria) are the dominant phyla, consistent with the dominant microbes assessed by 16S rRNA gene amplicon sequencing, suggesting that these MAGs may represent the dominating bacteria in the retrieved sediment core. In addition, Actinobacteriota (5/72), Acidobacteriota (4/72), and Gemmatimonadota (2/72) MAGs were also recovered. Finally, one MAG from each of Planctomycetota and Verrucomicrobiota was also recovered. Unfortunately, most of the recovered MAGs lack an identifiable 16S rRNA gene sequence, which prevented matching them with the OTUs obtained by the amplicon sequencing to track their vertical distributions throughout the core BRC2 with higher resolution. Nevertheless, the recovery of the genomes of microbes dominating in core BRC2 is helpful to elucidate their methylation profiles in the *in situ* environment.

**Fig 5:**
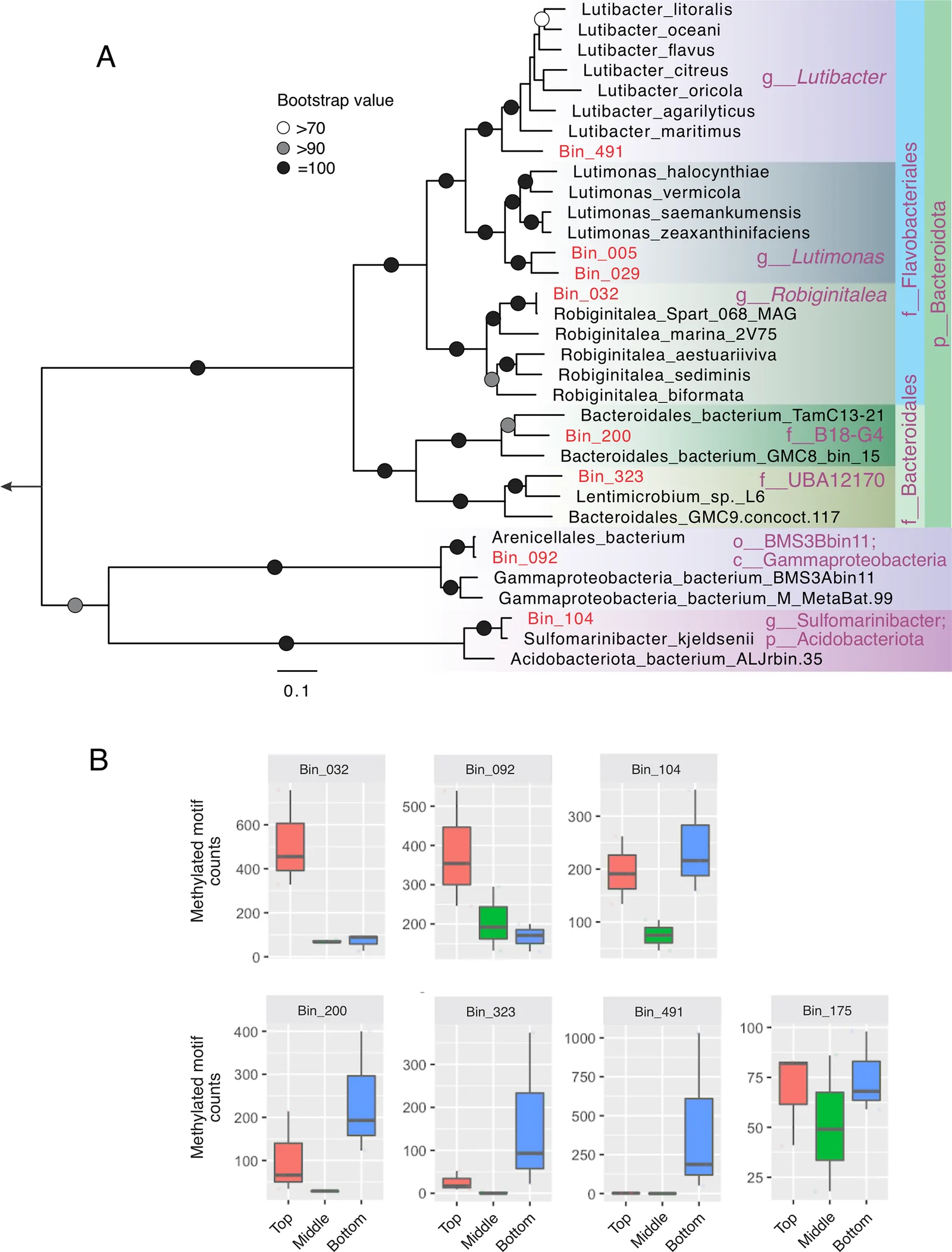
Phylogeny of selected MAGs and their varied total methylation counts with depth. (**A**) Maximum-likelihood phylogenetic tree based on the 120 bacterial single-copy proteins. The tree was inferred using IQ-TREE with LG + R5 as the best-fit evolutionary model and 1,000 ultra-fast bootstrap iterations. (**B**) Differentiated methylation counts of bacteria with depth. The three on the top row (i.e., Bin_092 of Gammaproteobacteria, Bin_032 of Bacteroidota, and Bin_104 of Acidobacteriota) showed a decreasing trend with depth. In contrast, the three bacterial MAGs on the bottom row (Bin_491, Bin_200, and Bin_323), all from the phylum Bacteroidota, showed an increasing trend with sediment depth. Finally, Bin_715, the only archaeal MAG recovered, showed comparable total methylation counts at the three examined zones.

Here, we focus on those having different methylation profiles along sediment depths. Two MAGs (Bin_005 and Bin_029) were classified as novel *Lutimonas* species, albeit with slight differences from the cultured one (Fig. 5A), which could represent the most abundant bacterium in core BRC2 (represented by OTU_7). In addition, Bin_491 represents a novel species within the *Lutibacter* genus (Fig. 5A). Moreover, Bin_032 is affiliated with the *Robiginitalea* genus. All these genera are within the Flavobacteriales order. In the Bacteroidales order, Bin_200 represents a new member in the f__B18-G4 family, while Bin_323 is in the f__UBA12170 family (Fig. 5A). Finally, Bin_092 represents a member in the o__BMS3Bin11 order of Gammaproteobacteria, while Bin_104 is closely related to *Ca*. Sulfomarinibacter kjeldsenii (Fig. 5A), an uncultured acidobacterium widespread in marine sediments (78). Recovering these individual genomes could enable us to recognize the epigenetic modification patterns of individual genomes along the sediment-forming process.

### Methylation of the dominant microbes in the sediment core

We selected the same nine sediment layers used for metagenome sequencing to analyze the methylation of the above-mentioned individual genomes, thereby covering the sediment-forming process as broadly as possible. In this study, we focused on cytosine and adenosine methylation motifs. We assessed the depth variation of the total counts of these two methylated motifs in the above-described MAGs of high completeness (>90% of genome completeness as assessed by CheckM2; Table S2), which are also among the dominant taxa.

These MAGs have different methylation counts across the examined sediment depths. For example, the total methylation counts of MAGs from three bacterial phyla (i.e., Bin_092 of Gammaproteobacteria, Bin_032 of Bacteroidota, and Bin_109 of Actinobacterota) showed a decreasing trend with depth (Fig. 5B). In contrast, three MAGs (Bin_491, Bin_200, and Bin_323), all from the phylum Bacteroidota, showed an increasing trend with sediment depth (Fig. 5B). Finally, Bin_715, the only archaeal MAG recovered from BRC2, showed comparable total methylation counts at the three examined zones (Fig. 5B). These results suggest that microbes may have different methylation responses to environmental changes associated with sediment burial, and this may be a deciding factor in a microbe’s survival during burial, as it has been suggested that any adaptation features to low energy life need to exist within a microbial genome at the time of burial (7). It is worth noting that the method we employed in this study cannot identify new methylation motifs, which seem to be widespread among uncultured microbes in the marine realm (e.g., reference 20). Therefore, more complementary methods are needed to comprehensively assess the methylation features of microbes adapting to the vast sediment environment.

### Conclusion and outlook

To study how microbes adapt to energy-limited conditions during the marine sediment burial process, we employed multiple complementary approaches to examine microbes in a sediment core formed in the laboratory with extremely high sedimentation rates. Our data suggested that microbial communities in the 23-cm sediment core showed minor vertical downcore variations, which may be due to the rapid sedimentation process that does not allow sufficient time for microbes to respond to environmental changes in the sediments. The dominant microbes, affiliated with the bacterial taxa Bacteroidota, Gammaproteobacteria, and Desulfobacterota, were mainly derived from the particulate matter in the incoming water. We also reconstructed genomes of the most dominant microbes in these taxa via metagenome sequencing and binning. Combining with the concurrent methylation sequencing data, our data suggest that despite the similar structure of the overall microbial community, some microbes in this sediment core exhibit different downcore methylation trends with depth, which may play distinct roles in enabling them to adapt to the sedimentary environment. Future work aiming to extensively examine the local geochemical conditions (e.g., the dominant organic matter mineralization pathways, respiration rates, and electron acceptor availabilities) would help to identify the major selection forces of microbes in the sedimentary environment.

## Data Availability

All sequence data from this project are available from the NCBI Short Read Archive under the BioProject accession number PRJNA1192328. The detailed accession numbers for individual sequencing data are provided in Table S1.

## SUPPLEMENTAL MATERIAL

Table S1 spectrum.03533-25-s0001.xlsx Amplicon sequencing data information.

Table S2 spectrum.03533-25-s0002.xlsx Genome quality summary.

ASM does not own the copyrights to Supplemental Material that may be linked to, or accessed through, an article. The authors have granted ASM a non-exclusive, world-wide license to publish the Supplemental Material files. Please contact the corresponding author directly for reuse.
